# Are there differences in affective temperaments between patients with Bipolar I and II disorder?

**DOI:** 10.1192/j.eurpsy.2022.572

**Published:** 2022-09-01

**Authors:** A. Rodríguez Rey, F. Piazza, L. Montejo, E. Jiménez, A. Martínez-Arán

**Affiliations:** Clinic Hospital, Psychiatry And Psychology, Barcelona, Spain

**Keywords:** TEMPS-A, temperament, bipolar disorder

## Abstract

**Introduction:**

Bipolar Disorder (BD) is a severe mental disorder with a high genetic load, in which is relevant to identify potential differences in affective temperaments between both diagnostic subtypes.

**Objectives:**

To find differences between BDI and BDII patients in affective temperaments evaluated by Temperament Evaluation of the Memphis, Pisa, Paris, and San Diego TEMPS-A.

**Methods:**

A sample of 407 euthymic patients with diagnosis of bipolar disorder type I (BDI= 307) or type II (BDII= 100) according to DSM-IV-TR criteria being age 18 or older was recruited from the Bipolar and Depressive Disorders Unit of the Hospital Clinic of Barcelona. Five affective temperaments were evaluated using the TEMPS-A. It was initially verified that the scores of these temperaments do not fulfil the assumption of normality by means of tests. Differences in means were estimated using Mann-Whitney U and Chi square tests (p <0.05) as appropriate, and ANCOVA controlling the effect of confounding variables.

**Results:**

Data revealed that patients with BD II had significantly higher scores in four affective temperaments: dysthymic, cyclothymic, irritable and anxious compared to BDI. After controlling the most relevant moderating variables, BDII patients continued to show higher scores in irritable temperament .

**Conclusions:**

BDII patients present a more irritable temperament than BDI (p=0,037), which can affect the course and management of the disease. It could be suggested that presenting higher scores of these temperaments could be associated with BDII and further studies are needed to replicate this finding since it might help the clinicians in early phases to guide in the diagnostic process.

**Disclosure:**

No significant relationships.

